# {4-Bromo-2-[2-(piperidin-1-ium-1yl)ethyl­iminometh­yl]phenolato}diiodido­zinc(II)

**DOI:** 10.1107/S1600536809037210

**Published:** 2009-09-19

**Authors:** Xue-Wen Zhu, Zhi-Gang Yin, Gang-Sen Li, Xu-Zhao Yang, Chun-Xia Zhang

**Affiliations:** a Key Laboratory of Surface and Interface Science of Henan, School of Materials and Chemical Engineering, Zhengzhou University of Light Industry, Zhengzhou 450002, People’s Republic of China

## Abstract

In the title complex, [ZnI_2_(C_14_H_19_BrN_2_O)], the Zn^II^ atom is four-coordinated by the imine N and phenolate O atoms of the Schiff base ligand and by two iodide ions in a distorted tetra­hedral coordination. In the crystal structure, mol­ecules are linked through inter­molecular N—H⋯O hydrogen bonds, forming chains running along the *b* axis.

## Related literature

For background to the chemistry of Schiff base complexes, see: Ali *et al.* (2008 ▶); Biswas *et al.* (2008 ▶); Chen *et al.* (2008 ▶); Darensbourg & Frantz (2007 ▶); Habibi *et al.* (2007 ▶); Kawamoto *et al.* (2008 ▶); Lipscomb & Sträter (1996 ▶); Tomat *et al.* (2007 ▶); Wu *et al.* (2008 ▶); Yuan *et al.* (2007 ▶). For related structures, see: Zhu (2008 ▶); Zhu & Yang (2008*a* ▶,*b* ▶,*c* ▶); Qiu (2006*a* ▶,*b* ▶); Wei *et al.* (2007 ▶); Zhu *et al.* (2007 ▶).
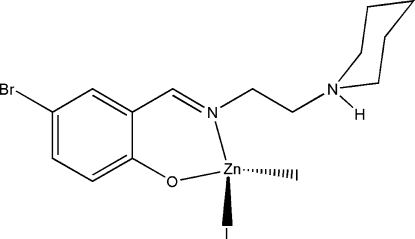


## Experimental

### 

#### Crystal data

[ZnI_2_(C_14_H_19_BrN_2_O)]
*M* *_r_* = 630.39
Monoclinic, 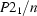

*a* = 10.470 (3) Å
*b* = 12.351 (3) Å
*c* = 15.426 (4) Å
β = 102.326 (3)°
*V* = 1948.8 (9) Å^3^
*Z* = 4
Mo *K*α radiation
μ = 6.48 mm^−1^
*T* = 298 K
0.17 × 0.15 × 0.15 mm

#### Data collection

Bruker APEXII CCD area-detector diffractometer
Absorption correction: multi-scan (*SADABS*; Sheldrick, 2004 ▶) *T*_min_ = 0.406, *T*_max_ = 0.443
14478 measured reflections
4193 independent reflections
2953 reflections with *I* > 2σ(*I*)
*R*_int_ = 0.053

#### Refinement

*R*[*F* ^2^ > 2σ(*F* ^2^)] = 0.044
*wR*(*F* ^2^) = 0.093
*S* = 1.03
4193 reflections
193 parameters
1 restraint
H atoms treated by a mixture of independent and constrained refinement
Δρ_max_ = 0.68 e Å^−3^
Δρ_min_ = −1.09 e Å^−3^

### 

Data collection: *APEX2* (Bruker, 2004 ▶); cell refinement: *SAINT* (Bruker, 2004 ▶); data reduction: *SAINT*; program(s) used to solve structure: *SHELXS97* (Sheldrick, 2008 ▶); program(s) used to refine structure: *SHELXL97* (Sheldrick, 2008 ▶); molecular graphics: *SHELXTL* (Sheldrick, 2008 ▶); software used to prepare material for publication: *SHELXL97*.

## Supplementary Material

Crystal structure: contains datablocks global, I. DOI: 10.1107/S1600536809037210/om2276sup1.cif

Structure factors: contains datablocks I. DOI: 10.1107/S1600536809037210/om2276Isup2.hkl

Additional supplementary materials:  crystallographic information; 3D view; checkCIF report

## Figures and Tables

**Table:** 

Zn1—O1	1.981 (4)
Zn1—N1	2.047 (4)
Zn1—I2	2.5428 (9)
Zn1—I1	2.5771 (9)

**Table:** 

O1—Zn1—N1	91.61 (16)
O1—Zn1—I2	114.27 (10)
N1—Zn1—I2	113.47 (12)
O1—Zn1—I1	109.08 (12)
N1—Zn1—I1	108.67 (12)
I2—Zn1—I1	116.86 (3)

**Table 2:** Hydrogen-bond geometry (Å, °)

*D*—H⋯*A*	*D*—H	H⋯*A*	*D*⋯*A*	*D*—H⋯*A*
N2—H2⋯O1^i^	0.90 (5)	1.85 (5)	2.745 (6)	176 (7)

Symmetry code: (i) 
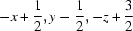
.

**Fig. 1.**
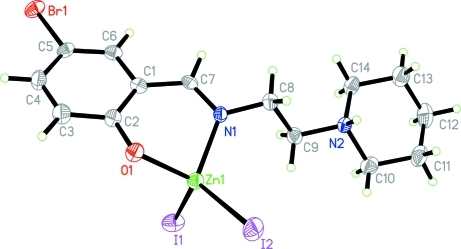
The molecular structure of the title complex, with ellipsoids drawn at the 30% probability level.

**Fig. 2.**
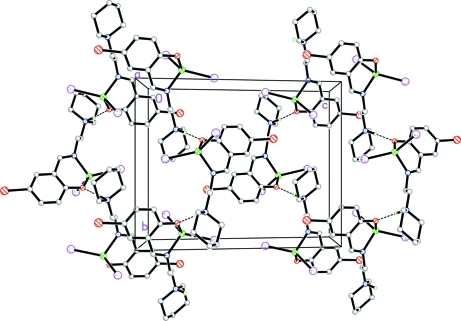
The crystal packing of the title complex.

## supplementary crystallographic information

### Comment

Schiff bases have widely been used as versatile ligands in coordination chemistry (Biswas *et al.*, 2008; Wu *et al.*, 2008; Kawamoto *et al.*, 2008; Ali *et al.*, 2008; Habibi *et al.*, 2007), and their metal complexes are of great interest in many fields (Chen *et al.*, 2008; Yuan *et al.*, 2007; Tomat *et al.*, 2007; Darensbourg & Frantz, 2007). Zinc(II) is an important element in biological systems and functions as the active site of hydrolytic enzymes, such as carboxypeptidase and carbonic anhydrase where it is in a hard-donor coordination environment of nitrogen and oxygen ligands (Lipscomb & Sträter, 1996). Recently, we have reported a few Schiff base zinc complexes (Zhu, 2008; Zhu & Yang, 2008*a*,*b*,*c*). In this paper, the title new zinc(II) complex, Fig. 1, is reported.

In the title complex, the Zn^II^ atom is four-coordinated by the imine N and phenolate O atoms of the Schiff base ligand, and by two iodide ions in a tetrahedral coordination. The coordinate bond lengths (Table 1) are typical and comparable to the corresponding values observed in the Schiff base zinc complexes we reported previously and other similar Schiff base zinc complexes (Zhu *et al.*, 2007; Wei *et al.*, 2007; Qiu, 2006*a*,*b*).

In the crystal structure, molecules are linked through intermolecular N—H···O hydrogen bonds, forming chains running along the *b* axis (Table 2, Fig. 2).

### Experimental

The Schiff base compound was prepared by the condensation of equimolar amounts of 5-bromosalicylaldehyde with 2-piperidin-1-ylethylamine in a methanol solution. The complex was prepared by the following method. To an anhydrous methanol solution (5 ml) of ZnI_2_ (31.9 mg, 0.1 mmol) was added a methanol solution (10 ml) of the Schiff base compound (31.1 mg, 0.1 mmol) with stirring. The mixture was stirred for 30 min at room temperature and filtered. Upon keeping the filtrate in air for a few days, colorless block-shaped crystals were formed.

### Refinement

H2 was located from a difference Fourier map and refined isotropically, with N—H distance restrained to 0.90 (1) Å. Other H atoms were placed in geometrically idealized positions and constrained to ride on their parent atoms, with C—H distances in the range 0.93–0.97 Å, and with *U*_iso_(H) = 1.2*U*_eq_(C).

### Crystal data

**Table:**

[ZnI_2_(C_14_H_19_BrN_2_O)]	*F*(000) = 1184
*M**_r_* = 630.39	*D*_x_ = 2.149 Mg m^−^^3^
Monoclinic, *P*2_1_/*n*	Mo *K*α radiation, λ = 0.71073 Å
Hall symbol: -P 2yn	Cell parameters from 2365 reflections
*a* = 10.470 (3) Å	θ = 2.5–25.1°
*b* = 12.351 (3) Å	µ = 6.48 mm^−^^1^
*c* = 15.426 (4) Å	*T* = 298 K
β = 102.326 (3)°	Block, colorless
*V* = 1948.8 (9) Å^3^	0.17 × 0.15 × 0.15 mm
*Z* = 4	

### Data collection

**Table:**

Bruker APEXII CCD area-detector diffractometer	4193 independent reflections
Radiation source: fine-focus sealed tube	2953 reflections with *I* > 2σ(*I*)
graphite	*R*_int_ = 0.053
ω scans	θ_max_ = 27.0°, θ_min_ = 2.1°
Absorption correction: multi-scan (*SADABS*; Sheldrick, 2004)	*h* = −13→12
*T*_min_ = 0.406, *T*_max_ = 0.443	*k* = −15→15
14478 measured reflections	*l* = −19→19

### Refinement

**Table:**

Refinement on *F*^2^	Primary atom site location: structure-invariant direct methods
Least-squares matrix: full	Secondary atom site location: difference Fourier map
*R*[*F*^2^ > 2σ(*F*^2^)] = 0.044	Hydrogen site location: inferred from neighbouring sites
*wR*(*F*^2^) = 0.093	H atoms treated by a mixture of independent and constrained refinement
*S* = 1.03	*w* = 1/[σ^2^(*F*_o_^2^) + (0.0326*P*)^2^ + 0.0119*P*] where *P* = (*F*_o_^2^ + 2*F*_c_^2^)/3
4193 reflections	(Δ/σ)_max_ = 0.001
193 parameters	Δρ_max_ = 0.68 e Å^−^^3^
1 restraint	Δρ_min_ = −1.09 e Å^−^^3^

### Special details

**Table:**

Geometry. All esds (except the esd in the dihedral angle between two l.s. planes) are estimated using the full covariance matrix. The cell esds are taken into account individually in the estimation of esds in distances, angles and torsion angles; correlations between esds in cell parameters are only used when they are defined by crystal symmetry. An approximate (isotropic) treatment of cell esds is used for estimating esds involving l.s. planes.
Refinement. Refinement of F^2^ against ALL reflections. The weighted R-factor wR and goodness of fit S are based on F^2^, conventional R-factors R are based on F, with F set to zero for negative F^2^. The threshold expression of F^2^ > 2sigma(F^2^) is used only for calculating R-factors(gt) etc. and is not relevant to the choice of reflections for refinement. R-factors based on F^2^ are statistically about twice as large as those based on F, and R- factors based on ALL data will be even larger.

### Fractional atomic coordinates and isotropic or equivalent isotropic displacement parameters (Å^2^)

**Table:**

	*x*	*y*	*z*	*U*_iso_*/*U*_eq_	
Zn1	0.16997 (7)	0.57708 (5)	0.70705 (4)	0.03725 (18)	
I1	−0.02704 (5)	0.68332 (4)	0.61843 (3)	0.05599 (16)	
I2	0.16625 (5)	0.53139 (4)	0.86722 (3)	0.05989 (16)	
Br1	0.46329 (7)	0.66143 (6)	0.32863 (4)	0.04966 (19)	
O1	0.3336 (4)	0.6472 (3)	0.6911 (2)	0.0409 (10)	
N1	0.2025 (4)	0.4456 (3)	0.6339 (3)	0.0316 (10)	
N2	−0.0218 (5)	0.2189 (4)	0.6686 (3)	0.0329 (10)	
C1	0.3224 (5)	0.5601 (4)	0.5493 (3)	0.0331 (13)	
C2	0.3580 (6)	0.6466 (4)	0.6099 (3)	0.0354 (13)	
C3	0.4260 (6)	0.7331 (5)	0.5832 (4)	0.0463 (16)	
H3	0.4494	0.7909	0.6221	0.056*	
C4	0.4595 (6)	0.7367 (5)	0.5032 (4)	0.0464 (15)	
H4	0.5063	0.7953	0.4883	0.056*	
C5	0.4238 (6)	0.6527 (5)	0.4440 (3)	0.0356 (14)	
C6	0.3578 (6)	0.5660 (4)	0.4664 (3)	0.0365 (14)	
H6	0.3356	0.5095	0.4261	0.044*	
C7	0.2634 (5)	0.4608 (4)	0.5703 (3)	0.0321 (13)	
H7	0.2697	0.4014	0.5343	0.039*	
C8	0.1621 (6)	0.3353 (4)	0.6490 (4)	0.0382 (14)	
H8A	0.2113	0.3096	0.7059	0.046*	
H8B	0.1814	0.2880	0.6032	0.046*	
C9	0.0187 (6)	0.3307 (4)	0.6481 (4)	0.0405 (14)	
H9A	−0.0013	0.3811	0.6916	0.049*	
H9B	−0.0305	0.3525	0.5901	0.049*	
C10	−0.1500 (6)	0.2221 (5)	0.6981 (4)	0.0511 (17)	
H10A	−0.2166	0.2535	0.6514	0.061*	
H10B	−0.1411	0.2680	0.7501	0.061*	
C11	−0.1929 (7)	0.1106 (6)	0.7199 (5)	0.0611 (19)	
H11A	−0.1316	0.0830	0.7714	0.073*	
H11B	−0.2780	0.1158	0.7352	0.073*	
C12	−0.2011 (7)	0.0321 (6)	0.6442 (5)	0.068 (2)	
H12A	−0.2200	−0.0400	0.6630	0.082*	
H12B	−0.2711	0.0533	0.5951	0.082*	
C13	−0.0735 (8)	0.0318 (5)	0.6150 (4)	0.0585 (19)	
H13A	−0.0804	−0.0155	0.5641	0.070*	
H13B	−0.0060	0.0030	0.6625	0.070*	
C14	−0.0340 (7)	0.1429 (5)	0.5910 (4)	0.0466 (16)	
H14A	−0.0989	0.1706	0.5414	0.056*	
H14B	0.0490	0.1388	0.5728	0.056*	
H2	0.038 (5)	0.197 (5)	0.716 (3)	0.080*	

### Atomic displacement parameters (Å^2^)

**Table:**

	*U*^11^	*U*^22^	*U*^33^	*U*^12^	*U*^13^	*U*^23^
Zn1	0.0429 (4)	0.0396 (4)	0.0303 (3)	−0.0049 (3)	0.0101 (3)	−0.0033 (3)
I1	0.0597 (3)	0.0710 (3)	0.0357 (2)	0.0164 (2)	0.0068 (2)	0.0024 (2)
I2	0.0858 (4)	0.0614 (3)	0.0341 (2)	−0.0066 (3)	0.0165 (2)	0.0058 (2)
Br1	0.0485 (4)	0.0681 (5)	0.0363 (3)	0.0073 (3)	0.0178 (3)	0.0093 (3)
O1	0.046 (3)	0.049 (2)	0.0271 (19)	−0.013 (2)	0.0072 (17)	−0.0100 (17)
N1	0.031 (3)	0.028 (2)	0.036 (2)	−0.003 (2)	0.008 (2)	0.0024 (19)
N2	0.034 (3)	0.034 (3)	0.030 (2)	−0.001 (2)	0.008 (2)	0.004 (2)
C1	0.030 (3)	0.034 (3)	0.032 (3)	0.002 (3)	0.000 (2)	−0.004 (2)
C2	0.036 (4)	0.040 (3)	0.029 (3)	−0.002 (3)	0.004 (2)	0.002 (2)
C3	0.062 (5)	0.047 (4)	0.029 (3)	−0.011 (3)	0.009 (3)	−0.007 (3)
C4	0.049 (4)	0.042 (4)	0.050 (4)	−0.009 (3)	0.015 (3)	0.005 (3)
C5	0.037 (4)	0.047 (4)	0.025 (3)	0.010 (3)	0.012 (2)	0.012 (2)
C6	0.043 (4)	0.040 (3)	0.026 (3)	0.006 (3)	0.006 (2)	−0.003 (2)
C7	0.037 (4)	0.028 (3)	0.030 (3)	0.001 (2)	0.004 (2)	−0.008 (2)
C8	0.038 (4)	0.037 (3)	0.039 (3)	−0.001 (3)	0.007 (3)	0.003 (3)
C9	0.040 (4)	0.036 (3)	0.045 (3)	0.004 (3)	0.009 (3)	0.007 (3)
C10	0.040 (4)	0.061 (4)	0.054 (4)	0.010 (3)	0.015 (3)	0.013 (3)
C11	0.047 (4)	0.066 (5)	0.075 (5)	−0.003 (4)	0.022 (4)	0.021 (4)
C12	0.061 (5)	0.061 (5)	0.078 (5)	−0.018 (4)	0.006 (4)	0.019 (4)
C13	0.075 (5)	0.050 (4)	0.053 (4)	−0.018 (4)	0.017 (4)	−0.010 (3)
C14	0.053 (4)	0.052 (4)	0.036 (3)	−0.014 (3)	0.014 (3)	−0.002 (3)

### Geometric parameters (Å, °)

**Table:**

Zn1—O1	1.981 (4)	C6—H6	0.9300
Zn1—N1	2.047 (4)	C7—H7	0.9300
Zn1—I2	2.5428 (9)	C8—C9	1.500 (8)
Zn1—I1	2.5771 (9)	C8—H8A	0.9700
Br1—C5	1.914 (5)	C8—H8B	0.9700
O1—C2	1.329 (6)	C9—H9A	0.9700
N1—C7	1.293 (7)	C9—H9B	0.9700
N1—C8	1.460 (6)	C10—C11	1.509 (9)
N2—C9	1.498 (7)	C10—H10A	0.9700
N2—C14	1.505 (7)	C10—H10B	0.9700
N2—C10	1.508 (7)	C11—C12	1.507 (10)
N2—H2	0.90 (5)	C11—H11A	0.9700
C1—C6	1.407 (7)	C11—H11B	0.9700
C1—C2	1.417 (7)	C12—C13	1.498 (10)
C1—C7	1.441 (7)	C12—H12A	0.9700
C2—C3	1.395 (8)	C12—H12B	0.9700
C3—C4	1.354 (8)	C13—C14	1.502 (8)
C3—H3	0.9300	C13—H13A	0.9700
C4—C5	1.380 (8)	C13—H13B	0.9700
C4—H4	0.9300	C14—H14A	0.9700
C5—C6	1.359 (8)	C14—H14B	0.9700
			
O1—Zn1—N1	91.61 (16)	N1—C8—H8B	109.5
O1—Zn1—I2	114.27 (10)	C9—C8—H8B	109.5
N1—Zn1—I2	113.47 (12)	H8A—C8—H8B	108.0
O1—Zn1—I1	109.08 (12)	N2—C9—C8	111.1 (4)
N1—Zn1—I1	108.67 (12)	N2—C9—H9A	109.4
I2—Zn1—I1	116.86 (3)	C8—C9—H9A	109.4
C2—O1—Zn1	117.4 (3)	N2—C9—H9B	109.4
C7—N1—C8	117.6 (4)	C8—C9—H9B	109.4
C7—N1—Zn1	118.0 (3)	H9A—C9—H9B	108.0
C8—N1—Zn1	124.4 (3)	N2—C10—C11	111.7 (5)
C9—N2—C14	112.8 (4)	N2—C10—H10A	109.3
C9—N2—C10	110.5 (4)	C11—C10—H10A	109.3
C14—N2—C10	109.1 (5)	N2—C10—H10B	109.3
C9—N2—H2	106 (5)	C11—C10—H10B	109.3
C14—N2—H2	113 (4)	H10A—C10—H10B	107.9
C10—N2—H2	106 (5)	C12—C11—C10	112.6 (6)
C6—C1—C2	118.6 (5)	C12—C11—H11A	109.1
C6—C1—C7	117.2 (5)	C10—C11—H11A	109.1
C2—C1—C7	123.9 (5)	C12—C11—H11B	109.1
O1—C2—C3	119.1 (5)	C10—C11—H11B	109.1
O1—C2—C1	123.3 (5)	H11A—C11—H11B	107.8
C3—C2—C1	117.5 (5)	C13—C12—C11	109.1 (6)
C4—C3—C2	122.8 (6)	C13—C12—H12A	109.9
C4—C3—H3	118.6	C11—C12—H12A	109.9
C2—C3—H3	118.6	C13—C12—H12B	109.9
C3—C4—C5	119.5 (6)	C11—C12—H12B	109.9
C3—C4—H4	120.2	H12A—C12—H12B	108.3
C5—C4—H4	120.2	C12—C13—C14	112.5 (6)
C6—C5—C4	120.3 (5)	C12—C13—H13A	109.1
C6—C5—Br1	120.2 (4)	C14—C13—H13A	109.1
C4—C5—Br1	119.4 (4)	C12—C13—H13B	109.1
C5—C6—C1	121.3 (5)	C14—C13—H13B	109.1
C5—C6—H6	119.4	H13A—C13—H13B	107.8
C1—C6—H6	119.4	C13—C14—N2	110.8 (5)
N1—C7—C1	126.8 (5)	C13—C14—H14A	109.5
N1—C7—H7	116.6	N2—C14—H14A	109.5
C1—C7—H7	116.6	C13—C14—H14B	109.5
N1—C8—C9	110.9 (4)	N2—C14—H14B	109.5
N1—C8—H8A	109.5	H14A—C14—H14B	108.1
C9—C8—H8A	109.5		

### Hydrogen-bond geometry (Å, °)

**Table:**

*D*—H···*A*	*D*—H	H···*A*	*D*···*A*	*D*—H···*A*
N2—H2···O1^i^	0.90 (5)	1.85 (5)	2.745 (6)	176 (7)

Symmetry codes: (i) −*x*+1/2, *y*−1/2, −*z*+3/2.
